# Safety profiles of terahertz scanning in ophthalmology

**DOI:** 10.1038/s41598-021-82103-9

**Published:** 2021-01-28

**Authors:** Yu-Chi Liu, Lin Ke, Steve Wu Qing Yang, Zhang Nan, Ericia Pei Wen Teo, Nyein Chan Lwin, Molly Tzu-Yu Lin, Isabelle Xin Yu Lee, Anita Sook-Yee Chan, Leopold Schmetterer, Jodhbir S. Mehta

**Affiliations:** 1 Singapore Eye Research Institute, The Academia, 20 College Road, Discovery Tower, Level 12, Singapore, 169856 Singapore; 2 Singapore National Eye Centre, Singapore, Singapore; 3 Ophthalmology and Visual Science Academic Clinical Research Program, Duke-NUS Medical School, Singapore, Singapore; 4 Institute of Materials Research and Engineering, Agency for Science, Technology and Research, Singapore, Singapore; 5 School of Chemical and Biomedcial Engineering, Nanyang Technological University, Singapore, Singapore; 6 Center for Medical Physics and Biomedical Engineering, Medical University of Vienna, Vienna, Austria; 7 Department of Clinical Pharmacology, Medical University of Vienna, Vienna, Austria; 8 Institute of Molecular and Clinical Ophthalmology, Basel, Switzerland

**Keywords:** Translational research, Medical imaging

## Abstract

Terahertz (THz) technology has emerged recently as a potential novel imaging modality in biomedical fields, including ophthalmology. However, the ocular biological responses after THz electromagnetic exposure have not been investigated. We conducted a rabbit study to evaluate the safety profiles of THz scanning on eyes, at a tissue, cellular, structural and functional level. Eight animals (16 eyes) were analysed after excessive THz exposure (control, 1 h, 4 h, and 1 week after continuous 4-h exposure; THz frequency = 0.3 THz with continuous pulse generated at 40 µW). We found that at all the time points, the corneas and lens remained clear with no corneal haze or lens opacity formation clinically and histopathologically. No thermal effect, assessed by thermographer, was observed. The rod and cone cell-mediated electroretinography responses were not significantly altered, and the corneal keratocytes activity as well as endothelial viability, assessed by in-vivo confocal microscopy, was not affected. Post-exposed corneas, lens and retinas exhibited no significant changes in the mRNA expression of heat shock protein (HSP)90AB1), DNA damage inducible transcript 3 (DDIT3), and early growth response (EGR)1. These tissues were also negative for the inflammatory (CD11b), fibrotic (fibronectin and α-smooth muscle actin), stress (HSP-47) and apoptotic (TUNEL assay) responses on the immunohistochemical analyses. The optical transmittance of corneas did not change significantly, and the inter-fibrillar distances of the corneal stroma evaluated with transmission electron microscopy were not significantly altered after THz exposure. These results provide the basis for future research work on the development of THz imaging system for its application in ophthalmology.

## Introduction

Terahertz (THz, 10^12^ Hz) waves are electromagnetic radiation. Recently, THz technology has emerged as a novel, noninvasive and noncontact imaging modality in biomedical fields. As water has a very high dielectric constant and is highly absorptive to THz spectrum, THz waves are therefore extremely sensitive to water content in tissue, and THz scanning systems have been applied to a variety of hydration-related diseases or conditions^1^. By sensing the changes of water content in the tissue, THz technology has been used to predict skin flap failure earlier than clinical diagnosis^2^, to early detect the teeth that are intact but present with caries lesion^3^, to early identify the features of skin cancer in the skin biopsy samples^4^, to help in the diagnosis of otitis media by early detection of the presence of pus^5^, and to potentially assess the physiological dynamics of the tear film in dry eye syndrome^6,7^

The cornea is a transparent and avascular tissue that consists of 78% water by volume^8^. The high water content of the cornea, the homogeneity of corneal stromal tissue, and the relative lack of physiological variations compared to other structures in the body, allow THz imaging to be a promising method for sensing corneal hydration level as THz waves are very sensitive to water^9^. Corneal edema results from various etiologies, including endothelial dystrophy, previous surgery such as cataract surgery or glaucoma filtrating surgery, trauma, toxicity or hypoxia^8,10^. For patients with corneal edema, measurement of the corneal hydrodynamics is important in monitoring the disease progression. However, the current imaging tools, such as anterior segment optical coherence tomography (ASOCT) or ultrasound pachymetry, can only measure corneal thickness as a surrogate evaluation for corneal edema^11–13^. By contrast, an absolute corneal stromal water content can be measured by THz scanning^14^. Brown and his colleagues reported that in a porcine eye model, there was an approximately linear relationship between the THz reflectivity and water concentration^1^. The same group further conducted a feasibility study on rabbits and found a positive correlation between the THz reflection point signal and the corneal hydration, as well as the central corneal thickness (CCT) measured by a pachymetry device^15,16^. Of note, although the changes in the corneal water concentration in the experiment was only a few percent, the THz system was able to detect the difference within this interval with statistical significance^15^. These pre-clinical results indicated the potential of the application of THz technique in assessing corneal edema in an objective manner.

As the wavelength of THz is non-ionizing, the scanning system has been considered biologically innocuous. The safety of THz scans has been previously studied in in-vitro models^17–19^. In-vitro experiments demonstrated that no significant effects on cell cycle kinetics and no discernable chromosomal DNA damage after THz exposure up to 84.8 mW/cm^2^ ^20^. Koyama et al. evaluated the in-vitro cellular effects on human corneal epithelial cells following exposure to 0.12 THz radiation at 5 mW/cm^2^ for 24 h, and no significant genotoxicity or morphological changes were observed^21^. However, for ocular tissue in the field of ophthalmology, the safety profiles of THz scanning have not been studied and have to be ascertained before its application in ophthalmology.

In this study, we aimed to investigate the safety profiles, including the ocular cellular and tissue responses, as well as structural and functional changes, following continuous THz exposure, using a rabbit model. To our knowledge, this is the first report comprehensively studying the biological effects of THz on eyes. The results would provide the necessary evidence for further clinical application of THz system in ophthalmology.

## Results

### Clinical evaluation with slit lamp biomicroscopy and fundus photography

**Figure 1:**
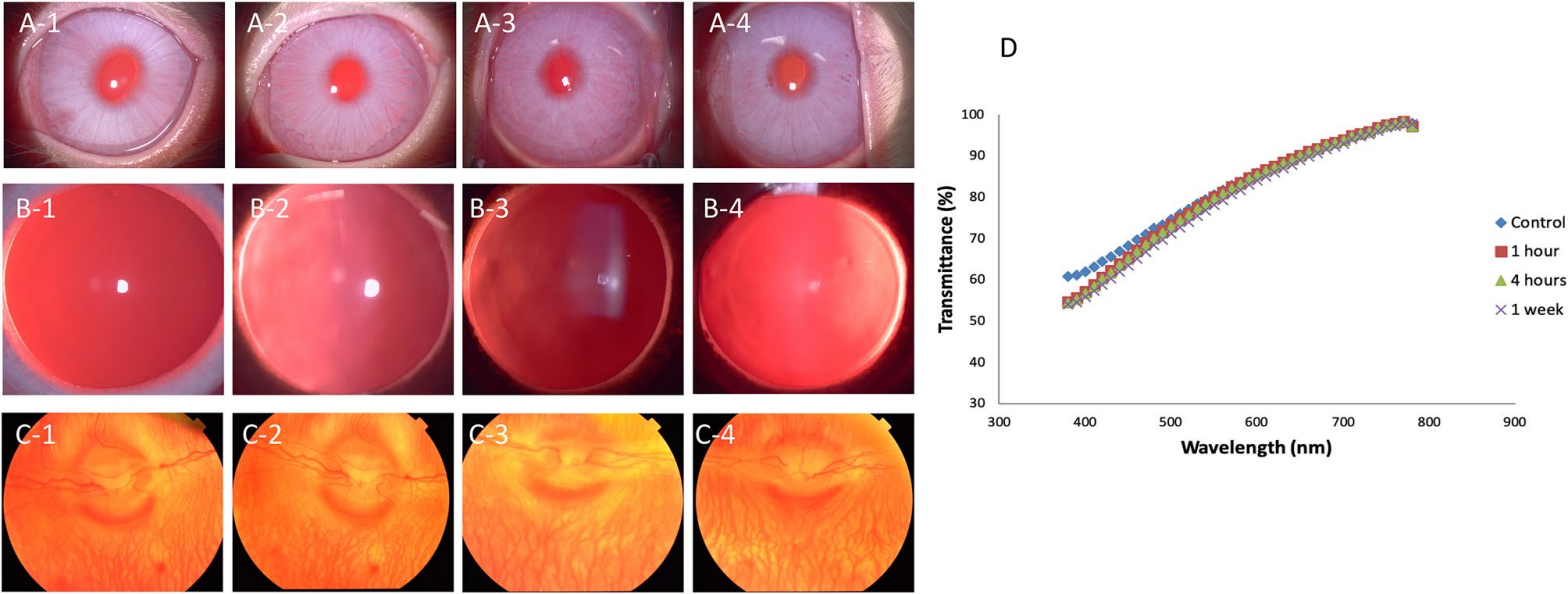
Clinical observation with slit lamp biomicroscopy (**A**,**B**) and fundus photography (**C**) for the control, 1 h, 4 h and 1 week after 4 h-exposure groups, respectively. Spectrum-wide transmittance trends of the corneas in different groups were also shown (**D**). The corneas remained clear without corneal haze formation (**A1**–**A4**), the lens were clear with no opacity (**B1**–**B4**), and there were no signs of vitritis, retinitis or optic neuropathy (**C1**–**C4**).

On the slit lamp evaluation, there was neither development of corneal haze and lens opacity, nor clinical signs of vitritis, retinitis and optic neuropathy, after 1-h and 4-h excessive THz exposure, or 1 week after 4-h exposure (Fig. 1A–C). The LOCS grade remained at 0 for the nuclear, cortical and posterior subcapsular opalescence in all the eyes. The mean spectral transmittance was 82.0 ± 1.9, 80.1 ± 2.0, 80.5 ± 1.7, and 79.5 ± 2.2, for the control, 1-h, 4-h, and 1-week groups, respectively (*P* = 0.51, *P* = 0.55, and *P* = 0.62 when comparing post-exposure values with controls; Fig. 1D).

### In vivo confocal micrographs analysis and ASOCT evaluation

**Figure 2:**
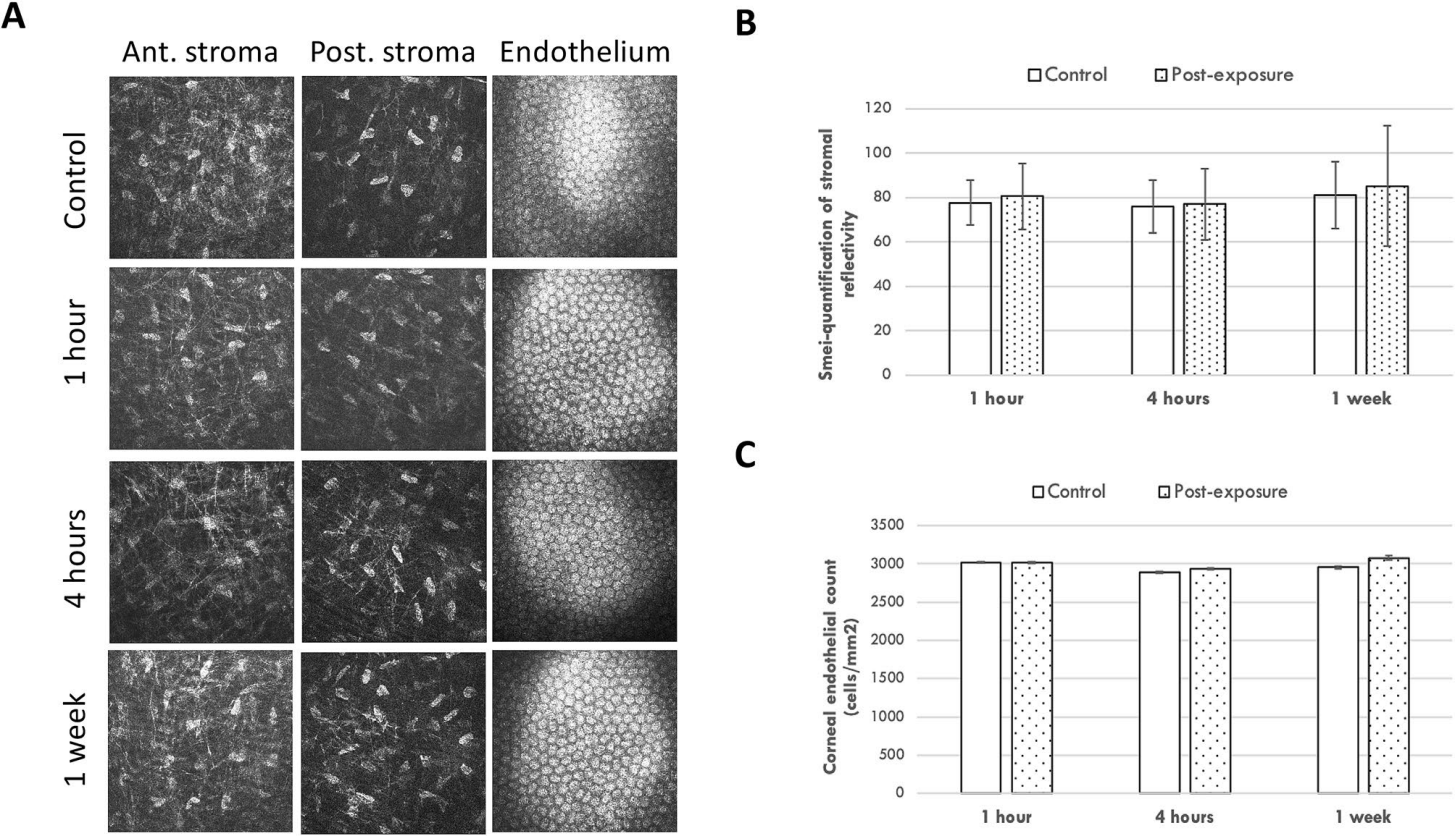
Representative IVCM micrographs at anterior stroma, posterior stroma and endothelial layer, after different periods of THz exposure (**A**). There were no significant changes in the mean intensity of stromal keratocytes reflectivity (**B**) and corneal endothelial density (**C**) when comparing to controls, at different time points. Error bars represent standard deviations.

On in-vivo confocal microscopy (IVCM) analysis, the stromal keratocytes appeared quiescent, and the keratocyte density at all the time points presented comparable to that in the controls (Fig. 2A). Semi-quantitative analysis of the intensity of the reflectivity revealed that the keratocyte reflectivity was not significantly different from that of the controls (mean gray value = 80.5 ± 2.8, 77.0 ± 3.7 and 85.2 ± 6.1, for the 1-h, 4-h, and 1-week time points, respectively; *P* = 0.79, *P* = 0.85, and *P* = 0.84 when comparing post-exposure values with controls; Fig. 2B). The corneal endothelium was healthy with no evidence of polymegathism and polymorphism (Fig. 2A). The corneal endothelial count was not affected, with the cell density at 3016 ± 119, 2937 ± 46, and 3078 ± 94 cells/mm^2^ for the 1-h, 4-h, and 1-week time points, respectively (*P* = 0.89, *P* = 0.77, and *P* = 0.83 when comparing post-exposure values with controls; Fig. 2C).

**Figure 3:**
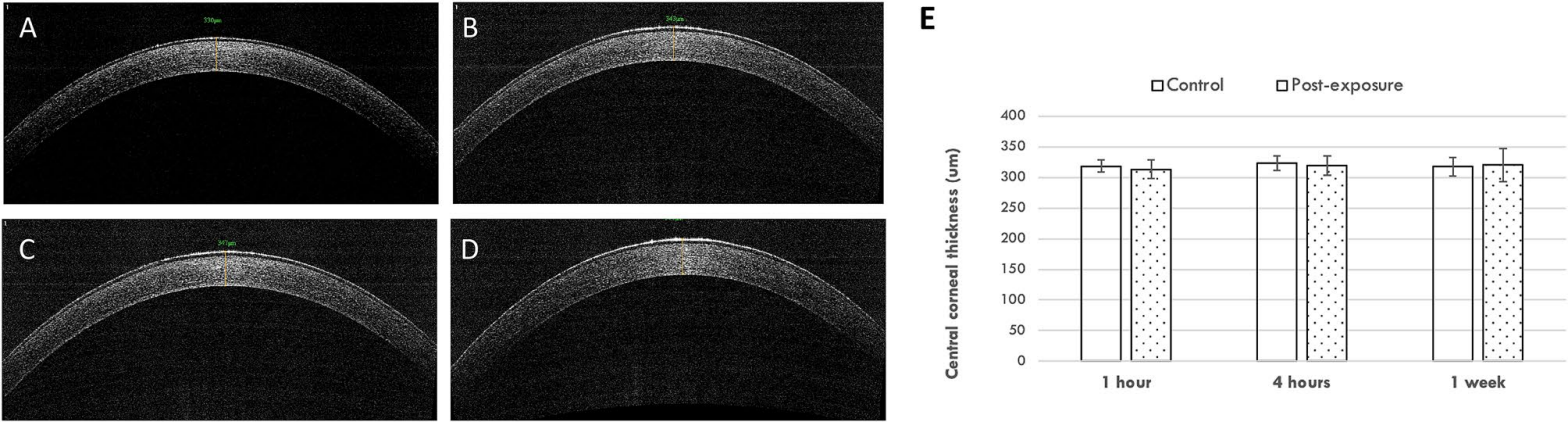
Representative ASCOT pictures for the control (**A**), 1 h (**B**), 4 h (**C**) and 1 week after 4 h-exposure groups (**D**). The bar graph showed that the CCT was at a consistent level over time after exposure (**E**). Error bars represent standard deviations.

On ASOCT evaluation, there was no abnormal hyper-reflectivity seen in the stroma. The CCT remained unchanged (313.3 ± 8.7, 319.3 ± 21.0 and 320.3 ± 15.5 µm, for the 1-h, 4-h, and 1-week time points, respectively; *P* = 0.89, *P* = 0.92, and *P* = 0.91 when comparing post-scanning values with control values; Fig. 3).

### Corneal temperature and ERG changes

**Figure 4:**
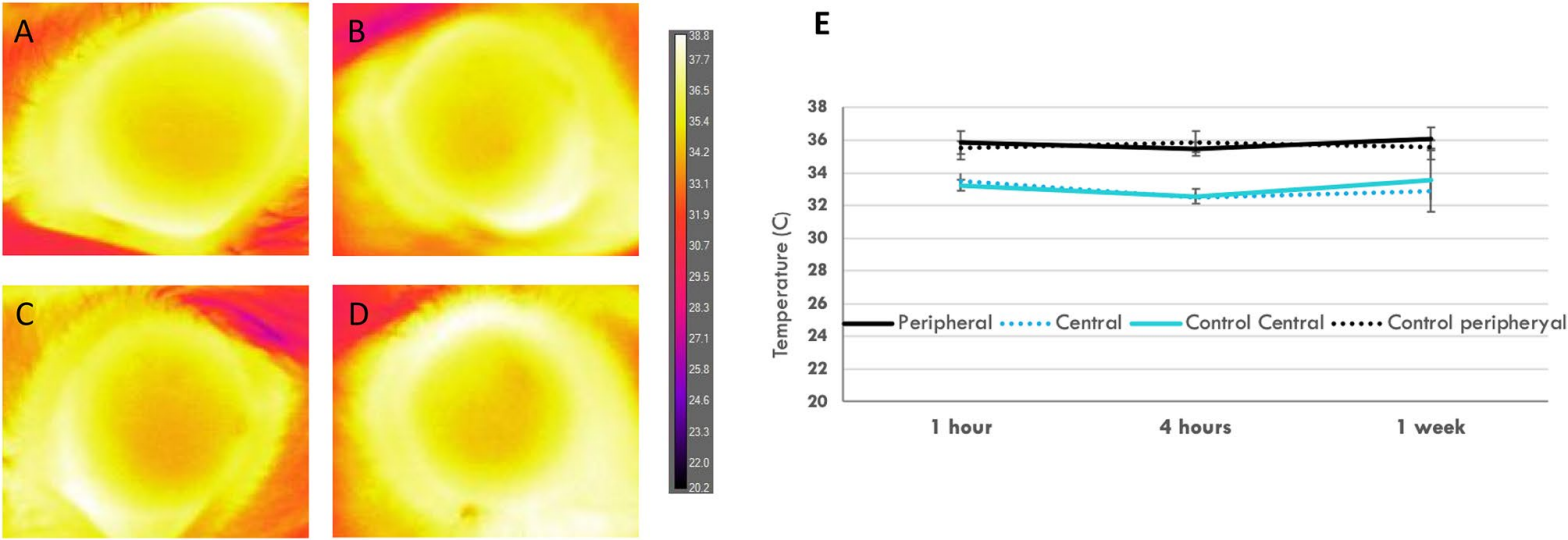
Representative thermographic maps showing the ocular surface temperature for the control (**A**), 1 h (**B**), 4 h (**C**) and 1 week after 4 h-exposure groups (**D**). The temperature was lower in the central area than that in the peripheral cornea, but no significant change was observed after different periods of exposure (**E**).

On the thermographic maps, the ocular surface temperature was lower in the central area than in the peripheral area. There were no significant changes in the central and peripheral temperature over the THz continuous scanning period (*P* = 0.69 when comparing different exposure time points). The central and peripheral temperatures, at all the time points, were comparable to those in the control group (all *P* > 0.05; Fig. 4).

**Figure 5:**
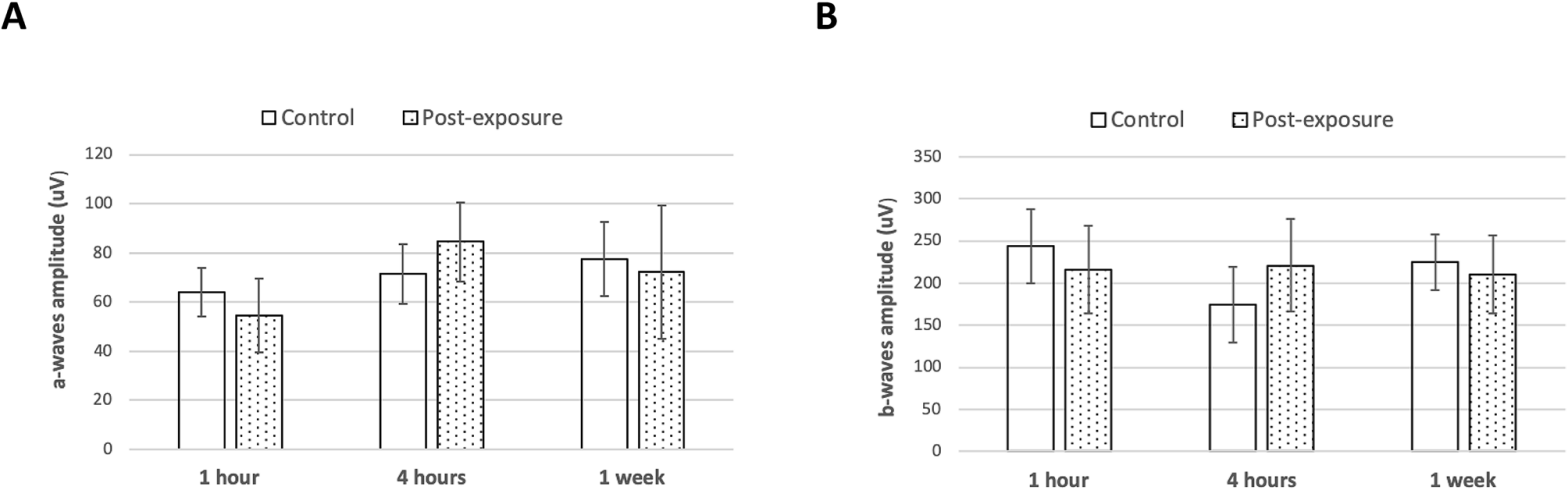
ERG responses after different duration of THz exposure. There were no significant changes in the scotopic a-wave (**A**) and b-wave (**B**) amplitudes after exposure compared to controls.

Retinal function evaluated by electroretinography (ERG) showed that the average scotopic a- and b-wave amplitudes obtained at 200 cd s/m^2^ were not significantly reduced in all the experimental groups (all *P* > 0.05 for a- and b-wave amplitudes at all the time points, when compared to controls; Fig. 5).

### Histological analysis and immunohistochemistry assays

**Figure 6:**
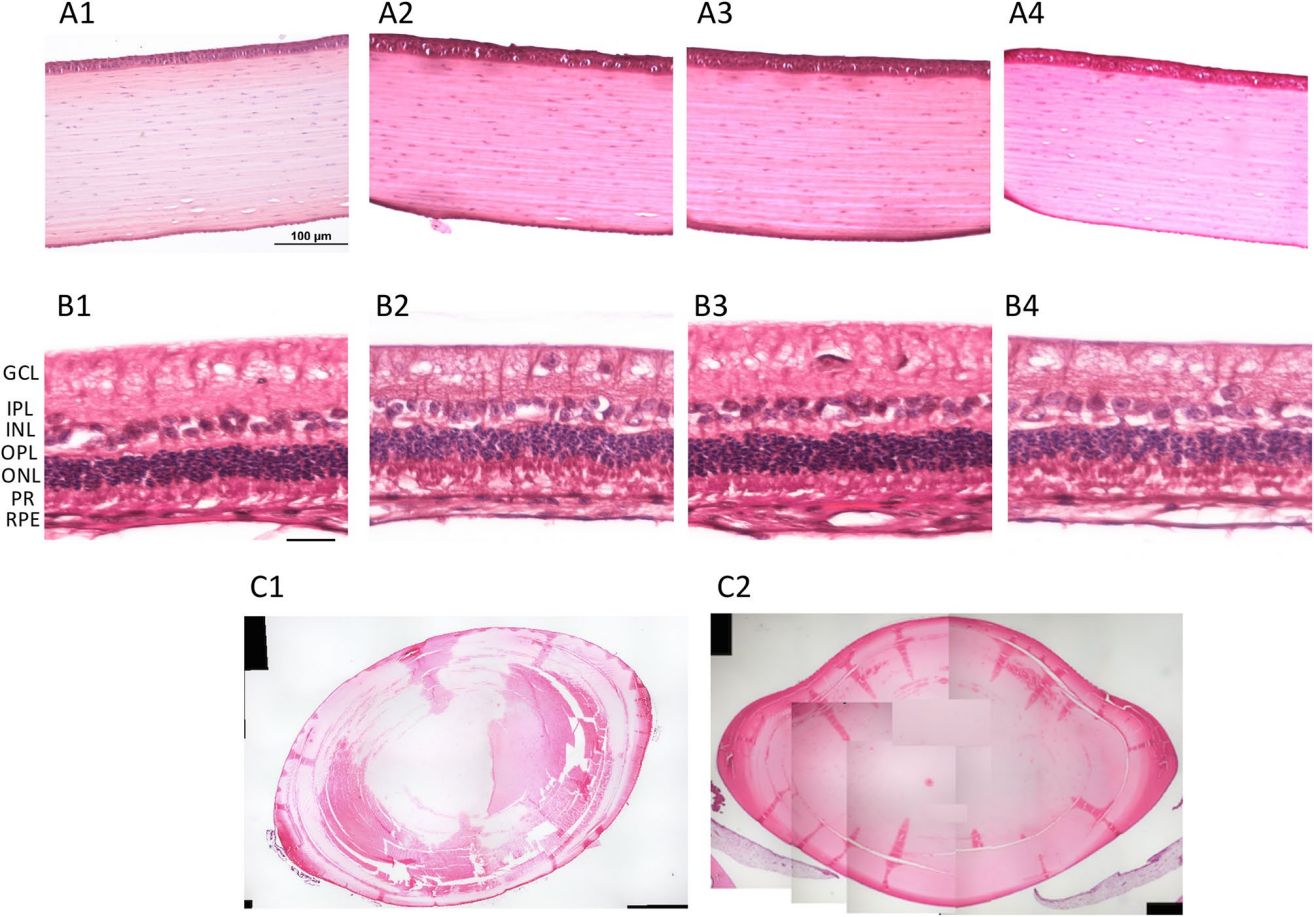
Histological sections with H&E staining presented that no inflammatory cell infiltrates or fibrotic reaction in the corneal stroma (**A1**–**A4**), and no retinal pathology such as gliosis, inflammation or degeneration of photoreceptors, were seen in the retinas (**B1**–**B4**), for the control, 1 h, 4 h and 1 week after 4 h-exposure groups, respectively. GCL: ganglion cell layer; IPL: inner plexiform layer; INL: inner nuclear layer; OPL; outer plexiform layer; ONL: outer nuclear layer; RPE: retinal pigment epithelium. After THz exposure, cataractogenesis was not detected. The crystalline lens fibers remained concentric and intact without disruption or liquefaction thus demonstrating no cortical cataract formation (note: artificial disruptions were seen due to processing artefacts). No increased coloration of the nuclear lens was seen thus excluding nuclear sclerosis (**C1**: control, **C2**: 1 week after 4-h exposure). Scale bar: 100 μm.

Histopathologically, no inflammatory cells or stromal fibrotic reaction were observed in corneas, and no signs of cell necrosis, gliosis, inflammation or degeneration of photoreceptors or neurons were seen in retinas in all eyes. The rings and parallel lens fibers were intact and parallel. No degeneration or vacuolation of the lens fibers, a characteristic of cortical cataract was seen. No accumulation of lens fibers or increased eosinophilic deposition of dense proteinaceous material, a pathological feature of nuclear cataract, was seen in the nucleus (Fig. 6). The histological sections were reviewed by an experienced ocular pathologist (A.S.Y.C) who had been masked to the experimental groups.

**Figure 7:**
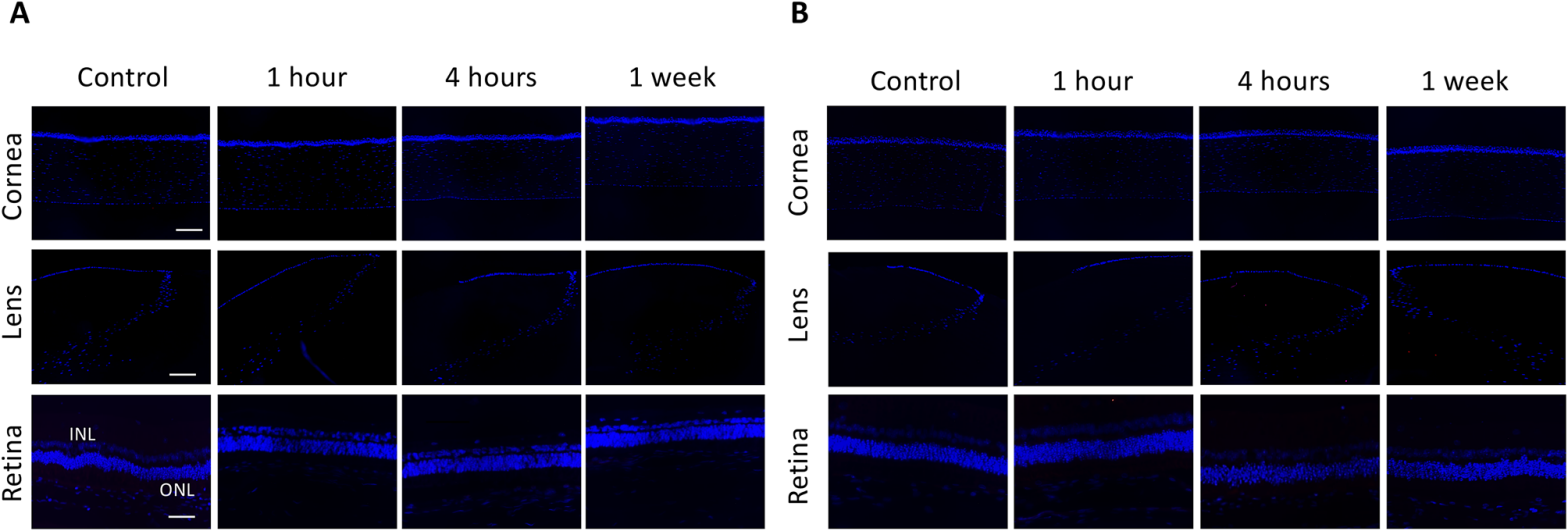
There was no expression of CD11b (**A**) and HSP-47 (**B**) in corneas, lens epithelium and retinas at different time points. IPL: inner plexiform layer; ONL: outer nuclear layer. Nuclei were counterstained with DAPI (blue). Scale bar 100 μm.

**Figure 8:**
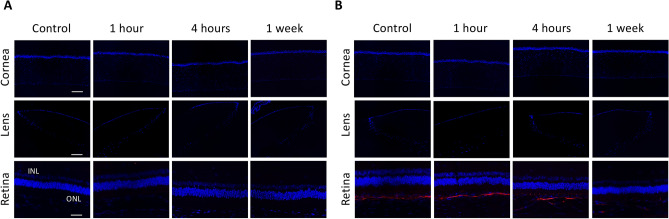
Immunohistochemical analysis on fibronectin showed negative staining in corneas, lens epithelium and retinas at all time points (**A**). Staining for α-SMA was present at Bruch’s membrane and sclera with a comparable extent in all eyes (**B**). IPL: inner plexiform layer; ONL: outer nuclear layer. Nuclei were counterstained with DAPI (blue). Scale bar 100 μm.

**Figure 9:**
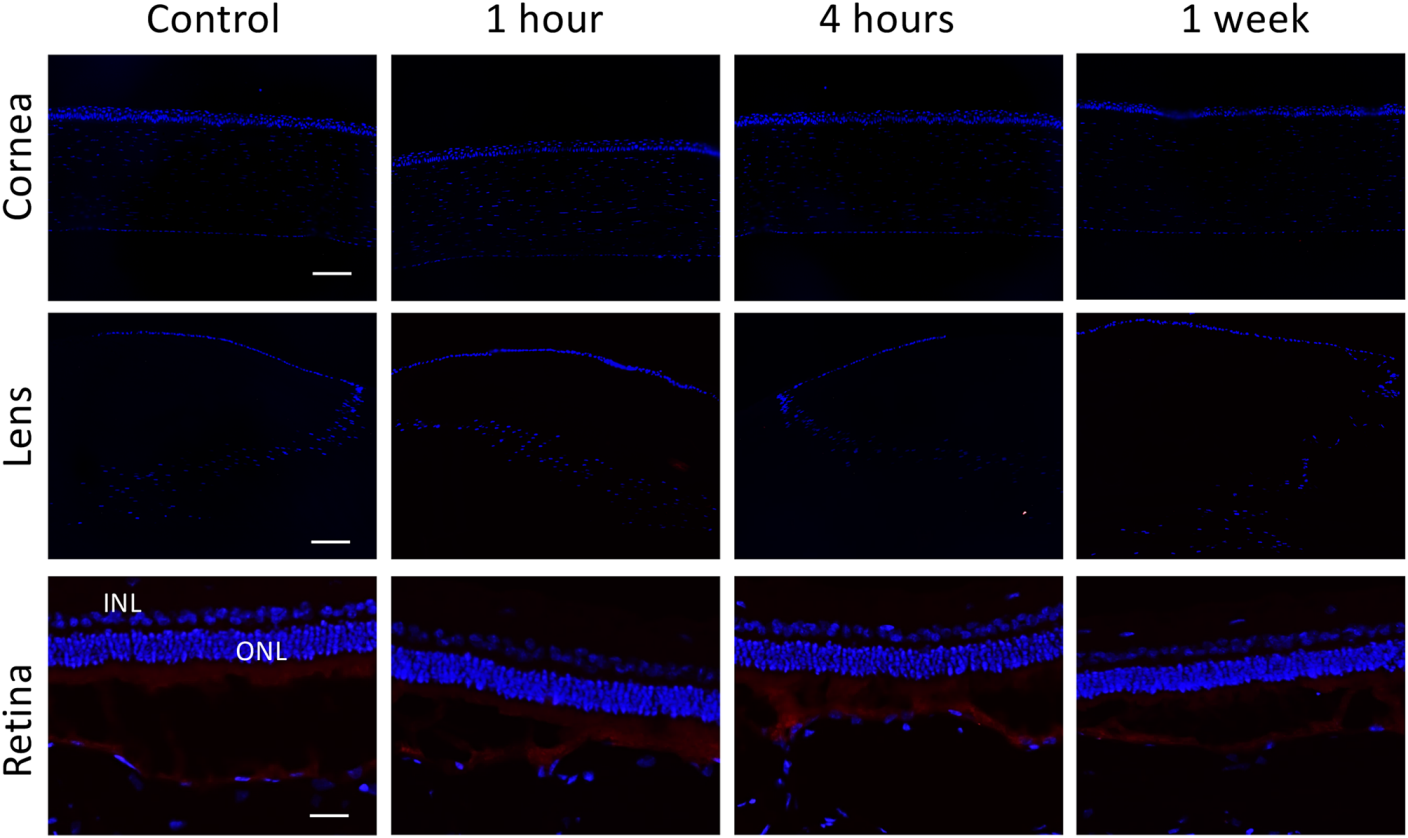
There were no TUNEL-positive staining cells at all time points, in corneas, lens epithelium and retinal layers. IPL: inner plexiform layer; ONL: outer nuclear layer. Nuclei were counterstained with DAPI (blue). Scale bar 100 μm.

There was no expression of CD11b and heat shock protein (HSP)-47, a macrophage marker in early inflammatory response and a collagen-specific stress protein marker, respectively, in corneas, lens epithelium and retinas in all eyes (Fig. 7). There was also no expression of fibronectin observed at all the time points. α-smooth muscle actin (α-SMA), a marker for myofibroblast transformation, was consistently present at Bruch’s membrane and sclera with no increase in the extent after THz exposure (Fig. 8). Apoptotic cells were not seen in corneas, lens epithelium, inner and outer nuclear layers of retinas at 1 h, 4 h and 1 week after THz radiation (Fig. 9).

### Corneal ultrastructure evaluated by TEM

**Figure 10:**
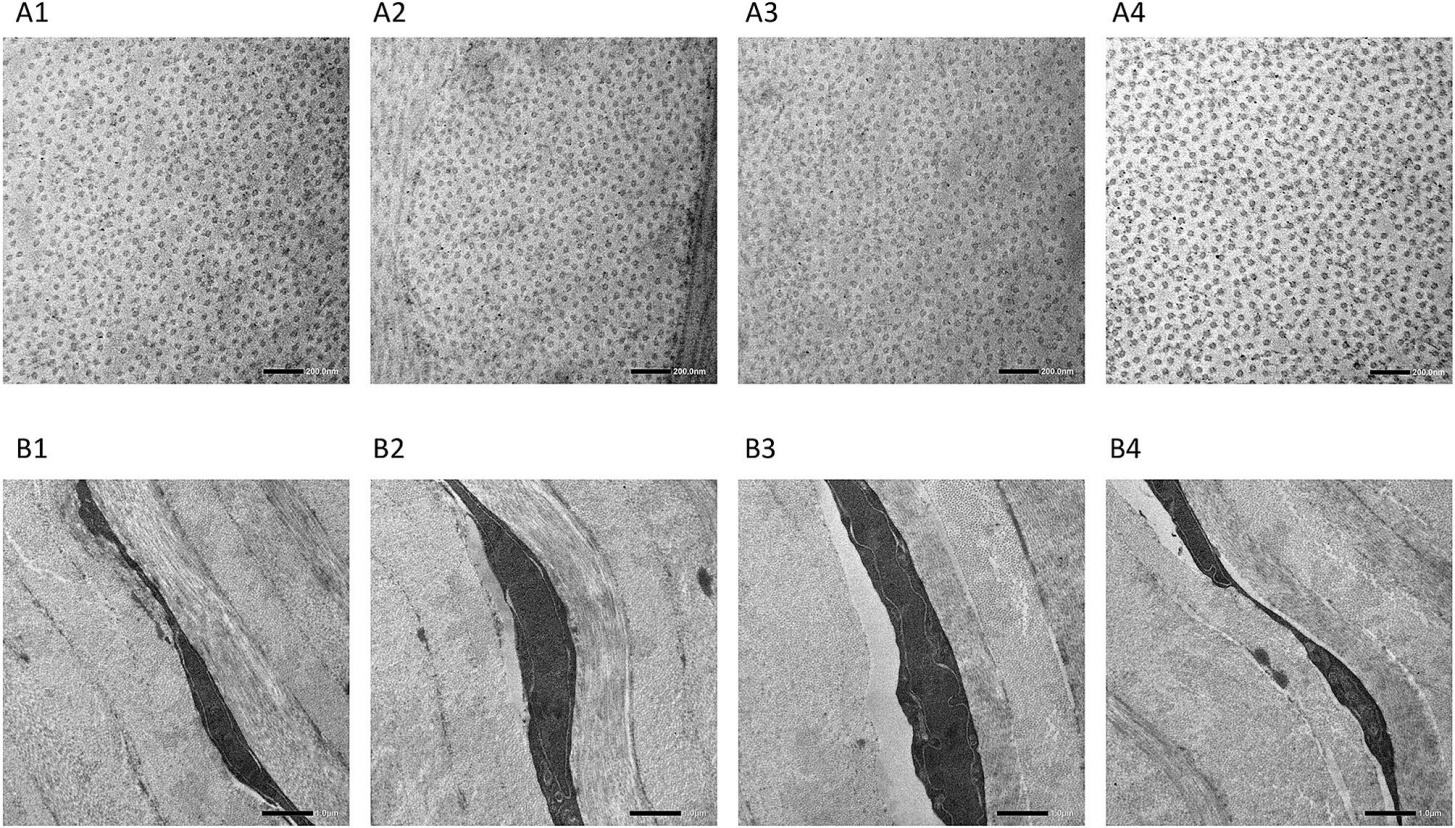
Transmission electron micrographs of the corneas showing transverse section of collagen fibrils (**A**, scale bar 200 nm) and keratocytes (**B**, scale bar 1 μm) for the control , 1 h , 4 h and 1 week after 4 h-exposure groups (A1–4 and B1–4, respectively).

The inter-fibillar distance was 60.7 ± 4.9, 50.6 ± 2.7, 66.5 ± 3.8, and 57.8 ± 3.1 pixel length, for the control, 1 h, 4 h and 1 week group respectively (*P* = 0.25, *P* = 0.29 and *P* = 0.33 when comparing the experimental groups to controls). The chromatin in the keratocyte nucleus was condensed in all the corneas, and no signs of cell necrosis, such as swelling nuclei, cytoplasmic vacuoles or irregular clumpings of chromatin, were observed (Fig. 10).

### mRNA expression and qRT-PCR

**Figure 11:**
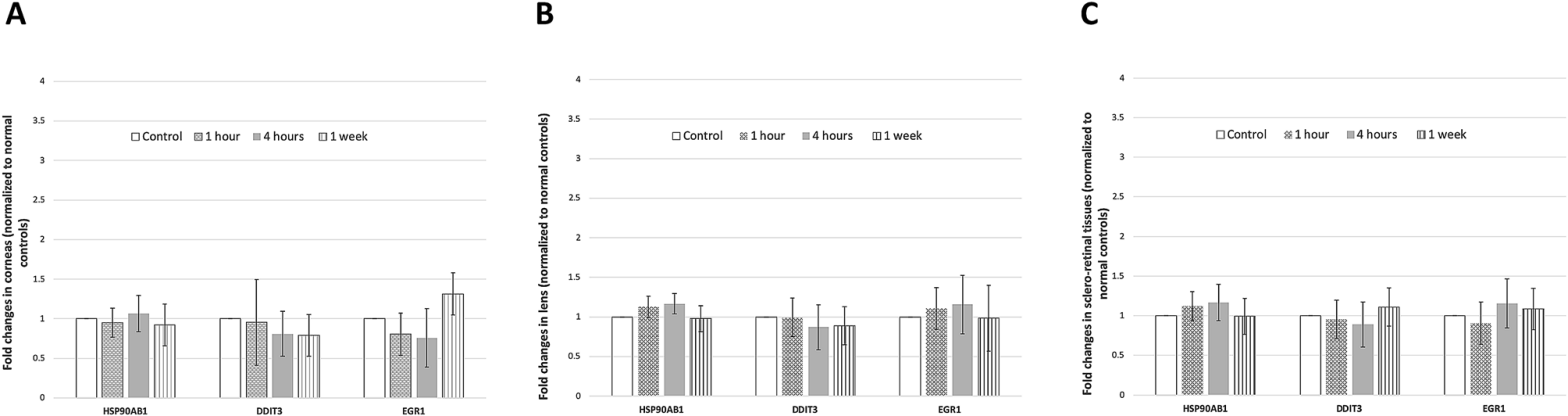
Gene expression in corneas (**A**), lens (**B**) and sclero-retinal tissues (**C**) measured for different groups using qRT-PCR. The mRNA expression fold values (2^−ΔΔCT^) were measured and normalized to that of the control group. There were no significant changes in the expression of HSP90AB1, DDIT3 and EGR1 in all the exposure groups.

The levels of mRNA expression of HSP90AB1, DDIT3, and EGFR1, which is a widespread heat-associated protein, a marker of cell stress response, and a transcriptional regulator, respectively, did not change significantly in the corneas, lens and scleral-retinal tissue, in the THz exposure groups, compared to the control group (all *P* > 0.05). The fold changes were at the range of 0.94–1.17, 0.79–1.11 and 0.75–1.31 for HSP90AB1, DDIT3, and EGR1 levels, respectively (Fig. 11).

## Discussion

In the present study, we demonstrated the biological responses, at tissue and cellular levels, after prolonged exposure to THz waves. With excessive exposure to THz up to 4 h, we did not observe detrimental effects on ocular tissue: the corneas and lens remained clear, the rod and cone cells- mediated ERG responses were not significantly altered, the thermal effect on ocular surface was not notable, and the corneal keratocytes activity, as well as endothelial viability, was not affected. The tissue reaction, including the inflammatory, apoptotic, fibrotic and stress responses, as well as the alternations in the corneal optical transmittance and ultrastructure, were not observed. These biological safety results are essential to pave the path for further potential clinical applications of THz scanning systems.

The application of THz sensing to the field of ophthalmology was first introduced in 2010^22^. Due to a very high dielectric constant and high sensitivity to changes of water content, THz imaging system has been reported as a potential non-contact and non-invasive tool to quantify corneal hydration level^22^. In an ex-vivo rabbit model, Elena et al. reported that an 1% decrease in the content of water mass in corneas led to a clearly detectable drop of the THz reflected signal by 13%, indicating good detection sensitivity^23^. Its high sensitivity to water molecules allows it to be a potential novel device to diagnose early water gradient changes, before measurable changes in corneal thickness by conventional devices such as ASOCT or ultrasonic pachymetry. The idea of using THz technology to generate hydration maps for an entire cornea was also proposed and tested, although many technical challenges related to the imaging resolution and imaging field have to be addressed^15,24^. These pre-clinical studies and pilot clinical study suggest that the THz-based system could be utilized as an independent or adjuvant diagnostic and monitoring tool, for patients with corneal edema or hydration-related eye diseases. As THz applications have been increasingly appearing, it is imperative to investigate the biological effects of THz exposure at standard-scanning usage levels.

The biological effects resulting from radiation depend on three factors: the type of radiation, the amount and the frequency of radiation, and the type of cells affected^25,26^. This can explain why there are some contradictory results concerning the biological effects of THz on different cells types. There was no genomic damage observed in in-vitro human skin cells after 2–8 h-THz exposure with the THz frequency up to 2.52 THz (power intensity 0.03–0.4 mW/cm^2^)^27^. An exposure of 0.12 THz radiation at 5 mW/cm^2^ for 24 h also did not induce genotoxicity, morphological changes, and HSP protein expression in in-vitro human corneal epithelial cells^21^. Similar safety was reported in blood samples from 9 healthy donors: no direct chromosomal damage and alteration of cell cycle kinetics on blood cells following 20 min and 0.12 THz exposure (intensity 1 mW/cm^2^)^20^. On the other hand, Alexandrov et al. reported that the expression of certain genes was affected in mouse mesenchymal stem cells after prolonged (9 h) and broad-band (10 THz) exposure^28^. This might be due to the nature of high radiosensitivity of stem cells^29^ and high-intensity exposure. The authors also concluded that the alteration of genes did not result from thermal effects as the increase in temperature was minimal, which is in agreement with the present study. The present study used a commercialized THz system in which the continuous pulse generated was at 40 µW and 0.3 THz. The exposure period in the experiments was prolonged, up to 4 h, which is much longer than the actual image acquisition time (within a minute). Moreover, as the biological expression after radiation can be immediate if the exposure is vast, or can be few days after the molecular absorption event (delayed onset)^30^, we also included a delayed time point at 1 week after exposure. On the molecular basis, we did not observe significant changes in the mRNA expression of HSP90AB1, DDIT3, and EGFR1 in our in-vivo experiments. Of note, previous studies used in-vivo experiment^20,21,27,28^, which were not taken into account physical barrier and buffer of the tissues. In normal eyes, the presence of tear film, aqueous humor and vitreous body protects the direct exposure of radiation.

The wavelength of THz is approximately 30 μm, which is short enough to provide reasonable resolution but long enough to prevent serious loss of signal due to scattering^31^. The wavelength is longer than that of infrared and ultraviolet (UV) light but is shorter than that of microwaves. It has been shown that an UV irradiation, at a wavelength of 300 nm, has a penetration depth of around 0.5 mm^32^, and therefore the penetration of THz waves are expected to be deeper than 0.5 mm. Hence we examined the biological effects down to the retinal level. Furthermore, THz waves belong to non-ionizing radiation, defined as the electromagnetic radiation that does not carry enough photon energy to ionize atoms or molecules. In contrast, ionizing radiation, such as X-rays or gamma-rays, has a higher frequency and shorter wavelength than non-ionizing radiation and can cause health hazards. These physical characteristics provide the background knowledge on the evaluation of its clinical applications in the aspect of safety.

The lens is one of the radiosensitive tissue in the human body^33^, and radiation is a well-known risk factor for cataract^10^. UV and infrared light are absorbed in the lens, and lens epithelial cell DNA is easily damaged by oxidative stress, direct photochemical action of radiation, or thermal damage resulting from high-frequency vibration of radiation, causing cataract^30,33^. Ionizing radiation, compared to non-ionizing radiation, has an even greater detrimental impact on the lens, hence radiological protection measures are required^33^. We did not observe any lens opacity, clinically or pathologically, after the THz exposure. The apoptotic cell death, the pathologic mechanism of cataractogenesis^34^, was not observed in the lens epithelium.

The cornea is at the anterior aspect of the eye and is highly exposed to irradiation. The wavelength of THz is close to that of infrared, and infrared devices have been safely applied in the diagnosis and treatment of several ocular surface diseases. Infrared meibography-ASOCT technology has been used to obtain the glandular architecture of Meibomian glands^35^. Infrared warm compression has been an effective treatment for patients with Meibomian gland dysfunction, by improving the release of meibum and tear stability^36^. With similar safety, we did not observe any side effects, such as stromal haze and negative impact on keratocytes or endothelium, evaluated clinically, optically, histologically and immunochemically, on the corneas after excessive THz exposure. On the other hand, radiation with longer wavelengths, such as microwaves, is associated with thermal effects, which can disrupt the extracellular matrix, change the inter-fibrillar distance, biomechanical property and transparency of the corneas^37^. Ionizing radiation, like gamma-rays, induce more detrimental effects, killing keratocytes and endothelium^38^. In the present study, the inter-fibrillar distances were not significantly altered after THz exposure, and no necrotic keratocytes were observed.

The irradiance transmitted to the retina is minimized by the radiation absorbance of the cornea, aqueous humor, lens and vitreous. The photoreceptors and retinal pigment epithelium (RPE) located in the posterior pole are more susceptible to radiation. Unprotected or prolonged exposure of UV light (UV-B particularly) results in photochemical damage in RPE and outer segment of photoreceptor^39^. Talebnejad et al. evaluated the effects of microwaves on rabbit’s retinas: there were no pathological changes on the histopathological sections, but the changes in the ERG responses were greater in the microwave groups than the sham group although not significant^40^. Similarly, our results revealed that no apoptotic, inflammatory, fibrotic or stress reaction was observed following prolonged THz exposure. However, the ERG evaluation did not have a consistent trend. The a- and b-waves were slightly and insignificantly lower in the exposure group than control group at 1 h. The waves were then returned to the control level at 4 h and 1 week.

There are several limitations in the present study. Firstly, as the wound healing process would be distinct 1 week after insult^41^, and the formation of cataract was reported a few days after radiation^30^, we set the last sacrificial time point at 1 week. Delayed onset consequences beyond one week will be investigated in future studies. Secondly, the ERG results were inconclusive, and this might be due to the inherently limited sensitivity of ERG on rabbits or small sample size.

In conclusion, we evaluated the biological responses of ocular tissue, ranging from the anterior segment to posterior segment, following excessive THz exposure. No adverse responses were observed from the tissue, cellular, structural or functional levels. These safety profiles provide favourable evidence and basis for further research work on the development and refinement of THz imaging system for its application in ophthalmology.

## Methods

### THz spectroscopy system

TERA K15 (Menlo Systems, GmbH, Germany) was used in this study. In the system, two femtosecond fibre lasers with 250 MHz repetition rate, 90 femtosecond laser pulses and approximately 1.56 μm central wavelength, were used to excite two photoconductive antennas: one was used as emitter and the other was used as a receiver. The THz emitter and receiver were based on the principle of a photoconductive switch. The THz pulse was generated with the coverage of bandwidth of 0.3–3 THz and 40 µW continuous power. The optical power of the lasers was approximately 30 mW with a pulse energy of approximately 0.3 nJ, which corresponded to 5 × 10^−13^ J for the THz radiation. The THz beam was focused with TPX lenses, and the wrist of focus beam was approximately 2 mm with maximum THz output at 0.3 THz. The repetition rates of both lasers were locked and stabilized by two synchronization electronic devices.

### Study animals and experimental groups

Sixteen 12- to 15-week-old New Zealand White rabbits (32 eyes) with 3–4 kg body weight were obtained from National University of Singapore and housed under standard laboratory conditions. All animals were treated according to the guidelines of the Animal Research: Reporting of In Vivo Experiments (ARRIVE guidelines, reference number 2017/SHS/1325). The protocol was approved by the Institutional Animal Care and Use Committee of SingHealth. The animals were randomly divided into 4 groups (n = 8 eyes of 4 animals for each): control, 1-h, 4-h and 1-week groups. In the 1-h and 4-h groups, the animals received prolonged THz radiation for 1 and 4 h, respectively, and then were euthanized under general anaesthesia by intracardiac injection of overdosed sodium pentobarbitone (Jurox, Rutherford, Australia). In the 1-week group, the rabbits were euthanized at 1 week after 4-h continuous THz exposure, in order to study any delayed tissue responses.

### Clinical evaluation

All the eyes underwent clinical evaluation by slit lamp biomicroscopy (Nikon FS-3V; Nikon, Japan), ASOCT (RTVue; Optovue, USA), IVCM (HRT3; Heidelberg Engineering GmbH, Germany), fundus photography (Micron IV fundus camera; Phoenix Research Laboratories, USA), ERG (E3; Diagnosys LLC, USA), and infrared ocular surface thermography (TG-1000; Tomey Corporation, Japan) under general anaesthesia at baseline and 1 h and 4 h after THz exposure, and 1 week after 4-h exposure. The lens opacity was graded with the Lens Opacities Classification system III (LOCS III)^42^.

For ASOCT evaluation, three high-resolution corneal cross-sectional scans (8 mm scan length, single scan mode) were obtained for each eye at each time point. The CCT was measured by an independent observer (NCL), and the average value was taken. For IVCM evaluation, the central aspect of the corneas was examined with a minimum of three z-axis scans, consisting of the entire corneal thickness. For each eye, three micrographs from anterior stroma (IVCM scanning depth < 160 μm), posterior stroma (scanning depth > 160 μm), and corneal endothelium, respectively, were selected. The gray values of reflectivity of six stromal scans were semi-quantified as described previously^43^, and the endothelial cells of 3 micrographs (frame area 400 μm × 400 μm) were counted, using Image J (National Institutes of Health, USA). The IVCM assessment was performed by two independent observers (YCL, NCL), and then the average value of micrographs was used. The fundus photographs were taken 30 min after instillation of dilation eye drops (1% tropicamide (Alcon, USA) and 2.5% phenylephrine (Bausch and Lomb, USA)). All the ERG recordings were performed in a dark room under dim red light illumination, as previously described^44^. Scotopic ERG responses were recorded across increasing light intensities from − 3.3 to 1.5 log cd s m^−2^ in 0.3-log-unit increments. Full-field ERG was recorded, and each response was the average of 3 trials. In addition, corneal temperature was evaluated with a contactless thermographer: the temperature of corneal surface was quantified with 28 equidistance test locations (each grid corresponded to 1.8 mm distance approximately)^45^, and we recorded the readings at the central cornea as well as 3.6 mm away from the center at each side (periphery).

### Histology and immunohistochemistry

The histologic and immunohistochemical analyses were performed as previously described^46,47^. In brief, sections of paraffin (5 µm thickness) embedded corneas, lens and retinas were stained with hematoxylin and eosin histochemistry and visualized under light microscopy (Axioplan 2; Carl Zeiss, Germany). For immunohistochemistry staining, sections were subjected to antigen retrieval in citrate buffer (pH = 6.0) for 20 min, and the slides were rinsed with phosphate-buffered saline (PBS). The slides were quenched with 10 mM ammonia chloride, followed by a blocking step for 1 h. They are then stained using the following primary antibodies: cellular fibronectin (Millipore, USA) diluted 1∶100; α-SMA (Dako Cytomation, Denmark) diluted 1∶50; HSP-47, Enzolife Sciences, Switzerland) diluted 1:200; CD11b (BD Pharmingen, USA) diluted 1:50, in the blocking solution. The secondary antibody was goat anti-mouse Alexa Fluor 488-conjugated (Invitrogen, USA). Slides were then mounted with UltraCruz mounting medium containing DAPI (Santa Cruz Biotechnology, USA) and were observed and imaged with a fluorescence microscope (Axioplan 2). To detect apoptotic cells, a fluorescence-based terminal deoxynucleotidyl transferase dUTP nick end labelling (TUNEL) assay (Roche Applied Science, USA) was used according to the manufacturer’s instructions.

### Optical transmittance measurements

Corneas from each group were placed in a 96-well plate, and 100 μL of wash buffer was added in to each well, together with an optical blank. Absorbance measurements were obtained using a Tecan Infinite M200 (Tecan, Männedorf, Switzerland). Absorbance, *A*, was measured over the wavelength range 380–780 nm at 1 nm intervals. Transmittance was calculated as T = 10^−A^, yielding transmittance of either the blank solution (*T*_*B*_) or of the samples (*T*_*B*+*S*_). Transmittance of the samples itself, *T*_*S*_, was then calculated as T_S =_ T_B+S_ + (1 − T_B_)^48^.

### Transmission electron microscopy (TEM) and quantitative real-time reverse transcription polymerase chain reaction (qRT-PCR)

TEM was performed with the protocol as we previously described^48^. In brief, corneas were fixed in 2% paraformaldehyde and 2% glutaraldehyde in PBS for one hour at room temperature and then cut into 1 mm^2^ small pieces, before being fixed for another 1 h and then washed 3 times for 5 min with PBS. Corneas were then fixed with 1% potassium ferrocyanide and 1% osmium tetroxide for 1 h and rinsed with distilled water. Subsequently, samples were dehydrated in a graded series of ethanol, and embedded in Araldite (Electron Microscopy Sciences, Pennsylvania*,* USA). The 70–90 nm ultra-thin sections were cut with an Ultramicrotome (C. Reichert Optische Werke AG, Austria) and were collected on copper grids, double stained with uranyl acetate and lead citrate for 8 min each, and then imaged on a JEM 1220 electron microscope (JEOL, Tokyo, Japan) at 100 kV. To evaluate the effects of THz on the collagen ultrastructure of corneas, two TEM images of transverse collagen fibrils from each quadrant was selected, and the fibril spacing was measured using Image J. The center-to-center interfibrillar distance (in terms of pixel length) was defined as the spacing between the reference fibril spot and its closest neighbors without other fibrils blocking in between^49^.

For qRT-PCR, the corneas, lens and sclero-retinal tissues were cut into small pieces and immediately transferred into chilled TRIzol reagent (Invitrogen, USA), followed by homogenization steps using sonication for 20 s at 20% power. Total RNA was extracted by the homogenized tissues using the PureLink Mini Kit (Ambion, Life Technologies, Carlsbad, CA, USA) according to manufacturer’s instructions. Total RNA was quantified using a NanoDrop ND-1000 UV–Vis Spectrophotometer (Thermo Scientific, USA). Total RNA (1 µg) from each sample was reverse transcribed into cDNAs using Superscript III (Invitrogen, USA) according to the manufacturer’s protocol. qRT-PCR was performed in 384-well plate in a total volume of 10 µL containing LightCycler 480 SYBR Green I Master (Roche, Switzerland), primers, PCR grade water, and cDNA.

Each pair of primers and samples were run in triplicate wells and were performed three times. The relative fold change was analyzed by the ΔΔC_T_ method. The expression level of each gene in the control samples was used for calibration. Threshold cycles (C_T_) were normalized to expression of the housekeeping gene glyceraldehyde-3-phosphate dehydrogenase (GAPDH)^50^: forward, 5′-GGG TGG TGG ACC TCA TGG T-3′, and reverse, 5′-CGG TGG TTT GAG GGC TCT TA-3′). ΔC_T_ in each sample was obtained by subtracting the C_T_ of GAPDH from the C_T_ of the targeted gene. ΔΔC_T_ of the samples for the 1-h, 4-h and 1-week groups was then calculated respectively by subtracting the ΔC_T_ of control samples from the ΔC_T_ of each THz-exposed samples. The fold change of the targeted gene in the THz-exposed samples compared with the controls was determined as 2^−ΔΔCT^. Gene specific primers were selected (PRIMER BLAST, NIH) as follows: HSP90AB1 (Heat shock protein 90 kDa alpha family class B member 1): forward, 5′-ATG ACT GGG AGG ACC ACT TG-3′, and reverse, 5′-GGG ATG AAA AGC AAA GCC CTG-3′; DDIT3 (DNA damage inducible transcript 3): forward, 5′-CTG TCC GTG TCC CCC AAG AT-3′, reverse, 5′-GGA GAG AGC GGT GCT TGC TA-3′; EGR1 (Early growth response 1): forward, 5′-CTA CGA GCA CCT GAC CGC A-3′, reverse, 5′-AGG GTG TTG CCA CTG TTG GG-3’.

### Statistical analysis

All data were expressed as mean ± standard deviation. Statistical comparisons among the data of different exposure time points were performed using a Friedman test. Comparisons between post-exposure values and controls were carried out with a Mann–Whitney U test. Statistical analyses were performed using STATA software (version 13, STATACrop, College Station, TX). *P* values less than 0.05 were considered statistically significant.
